# High-resolution ultrasound changes of the vagus nerve in idiopathic Parkinson’s disease (IPD): a possible additional index of disease

**DOI:** 10.1007/s10072-021-05183-5

**Published:** 2021-04-05

**Authors:** F. Sartucci, T. Bocci, M. Santin, P. Bongioanni, G. Orlandi

**Affiliations:** 1 Department of Clinical and Experimental Medicine, Cisanello Neurophysiopathology Unit, University of Pisa, School of Medicine, Via Paradisa, n. 2, I 56124 Pisa, Italy; 2 Integrated Care Department Medical Specialty, AOUP, Pisa, Italy; 3 Neuroscience Institute, CNR, Pisa, Italy; 4 Don Carlo Gnocchi Foundation, Massa, Italy; 5 “Aldo Ravelli” Center for Neurotechnology and Experimental Brain Therapeutics, Department of Health Sciences, International Medical School, University of Milan, Milan, Italy; 6 Department of Clinical and Experimental Medicine, Neurorehabilitation Unit, University Medical School, Pisa, Italy; 7 Severe Acquired Brain Injuries Unit, Cisanello University Hospital, Pisa, Italy; 8 Department of Clinical and Experimental Medicine, Neurology–Neurophysiopathology Unit, University of Pisa, School of Medicine, Pisa, Italy

**Keywords:** Vagus nerve, Parkinson’s disease, High-resolution ultrasounds (HRUS), Atrophy, Axonal degeneration, Neuropathy

## Abstract

**Background and rationale:**

Histopathological studies revealed degeneration of the dorsal motor nucleus of the vagus nerve (VN) early in the course of idiopathic Parkinson’s disease (IPD). Degeneration of VN axons should be detectable by high-resolution ultrasound (HRUS) as a thinning of the nerve trunk. In order to establish if the VN exhibits sonographic signs of atrophy in IPD, we examined patients with IPD compared with age-matched controls.

**Material and methods:**

We measured the caliber (cross-sectional area, CSA) and perimeter of the VN in 20 outpatients with IPD (8 females and 12 males; mean age 73.0 + 8.6 years) and in age-matched controls using HRUS. Evaluation was performed by blinded raters using an Esaote MyLab Gamma device in conventional B-Mode with an 8–19 MHz probe.

**Results:**

In both sides, the VN CSA was significantly smaller in IPD outpatients than in controls (right 2.37 + 0.91, left 1.87 + 1.35 mm^2^ versus 6.0 + 1.33, 5.6 + 1.26 mm^2^; *p* <0.001), as well as the perimeter (right 5.06 + 0.85, left 4.78 + 1.74 mm versus 8.87 + 0.86, 8.58 + 0.97 mm; *p* <0.001). There were no significant correlations between VN CSA and age, the Hoehn and Yahr scale, L-dopa therapy, and disease duration.

**Conclusion:**

Our findings provide evidence of atrophy of the VNs in IPD patients by HRUS. Moreover, HRUS of the VN represent a non-invasive easy imaging modality of screening in IPD patients independent of disease stage and duration and an interesting possible additional index of disease.

## Introduction

Idiopathic Parkinson’s disease (IPD) is known to be pathologically characterized by a progressive neuronal loss in the substantia nigra (SN) and pars compacta and aggregation of the α-synuclein protein that accumulates in Lewy bodies (LB) [1]. LB are highlightable histopathologically in many parts of the brain of IPD patients [2, 3], including the SN, locus coeruleus, basal nucleus of Meynert, cerebral cortex, and all cranial nerves nuclei, especially the dorsal motor vagal nucleus (DMVN) [1]. The accumulation of α-synuclein aggregates in some vagus nerve (VN) nuclei can be present even at earliest stages of IPD [4–6]. In addition, some authors, on the base of studies conducted on the topographic distribution of α-synuclein, have hypothesized that the neuropathological process leading to IPD may start in the gastroenteric nervous system and spread centrally via the VN to the lower brainstem [7–9], although this gut–brain transmission scenario still remains highly controversial [10, 11], even if it has opened new horizons of research [11]. The degree of arrival at the cerebral cortex is responsible for damage and cognitive reserve loss [12] and the consequent polymedication use [13] in IPD. Furthermore, autonomic dysfunction is present in the disease [14] and may precede the occurrence of the cardinal motor symptoms by many years [8, 15]. It involves the gastrointestinal tracts, which receive parasympathetic input via VN, and is responsible of constipation, gastroparesis, or nausea [8, 16]. From all these considerations originates the interest for the VN and its structural integrity in IPD.

Neurodegenerative processes involving neural cell bodies are followed by degeneration of their axons [17]; moreover, in IPD being involved the DMVN, it can be expected the VN to be thinner compared with controls. However, morphological changes related to axonal degeneration are subtle and hard to be detected in vivo. Mild-to-moderate atrophy of the VN has been detected in diabetic neuropathy [18, 19], and ALS [20] and reference value are available in [19, 21–24].

Few studies used HRUS to detect possible atrophy or not of the VN in IPD patients, with non-unique and definitive results. Some authors reported evident atrophy [15, 25, 26], whereas others did not [27, 28]. Therefore, the questions remain until now controversial and require further investigations. To unravel the hypothesis and answer the question whether IPD patients have or do not have VN atrophy and since HRUS represent a valuable tool to investigate the nerve trunks, we assessed sonographically VN integrity in IPD compared to age-matched healthy controls.

## Methods

### Study sample

We recruited 20 outpatients with a clinical and instrumental IPD, diagnosed according to the criteria of the UK British Brain Bank [29] and successive revision [30]. As supportive criteria to exclude other additional central nervous system pathologies, all cases had undergone previously at the time of diagnosis, CT and MRI scan, PET and SPECT examination, and eventually acute challenge test with levodopa, essential requirements for inclusion in the study. We excluded cases with neuroimaging signs of other central nervous system pathology, or systemic disease, like diabetes mellitus, vascular disease, and peripheral neuropathy.

The sample included 8 females and 12 males, mean age 73.0 + 8.6 years, mean disease duration 10.1 + 7.8 years (range 2–20 years); all patients were taking L-dopa therapy alone or together with dopamine agonists.

Control data for the VN parameters were obtained from volunteers, recruited among the staff of the department, colleague, and people who accompanied the subjects to outpatient visits, 10 females and 10 males, mean age 65.2 + 10.3 years. The demographic data of the two groups and the main clinical features of the patients are summarized in Table 1. There were no significant differences in age, sex, race, weight and height, or body mass index (BMI), between the two samples.

**Table 1:** Summary of demographic data and main clinical characteristic of both controls and patients with idiopathic Parkinson’s disease, enrolled in the study

	Controls (*n* = 20)	IPD patients (*n* = 20)	*p* value
Sex (f/m)	10 f/10 m	8 f/12 m	
Age (years)	65.2 + 10.3	73.0 + 8.6	n.s.
Height (cm)	169.89 + 7.10	170.1 + 5.01	n.s.
Weight (kg)	71 + 16.9	72.9 + 10.8	n.s.
Body mass index (BMI)	24.47 + 4.95	25.06 + 5.89	n.s.
IPD duration (years)		10.1 + 7.8 (range 2–20)	
L-dopa therapy		20 (100%)	
Other therapy (dopamine agonists)		18 (90%)	

*IPD*, idiopathic Parkinson’s disease; *f*, female; *m*, male

### Clinical assessment

All participants before inclusion underwent a profound physical examination to confirm the diagnosis, encompassing onset, course, disease duration, and severity by two of the authors (PB and BT). The Hoehn and Yahr staging [31] was employed to assess the disease severity. Patients were characterized by a moderate but clear motor impairment (mean Hoehn and Yahr stage 2.5 + 1)

A possible polyneuropathy was preliminary ruled out by means of nerve conduction studies and ultrasonography of tibial and sural nerve, as elsewhere reported [19].

The study was preliminarily approved by the local ethics committee of Azienda Ospedaliero-Universitaria in Pisa, Italy. All participants gave a written informed consent prior to be enrolled in the study, according to the Declaration of Helsinki and its later amendments.

### High-resolution ultrasonography

A single, study-blind sonographer (FS) performed all HRUS studies on a separate day from that of the clinical evaluation and enrollment of cases. The acquisition of ultrasound images was performed using an 8–19 MHz linear array transducer with an Esaote MyLab Gamma device (Esaote, Genova, Italy) in conventional B-mode [19]; cross-sectional area (CSA), perimeter, echogenicity, and fascicular structure were considered and measured either on the right and left side. The patients and controls were scanned by the same author at the Neurophysiopathology Section of the Department of Clinical and Experimental Medicine.

**Fig. 1:**
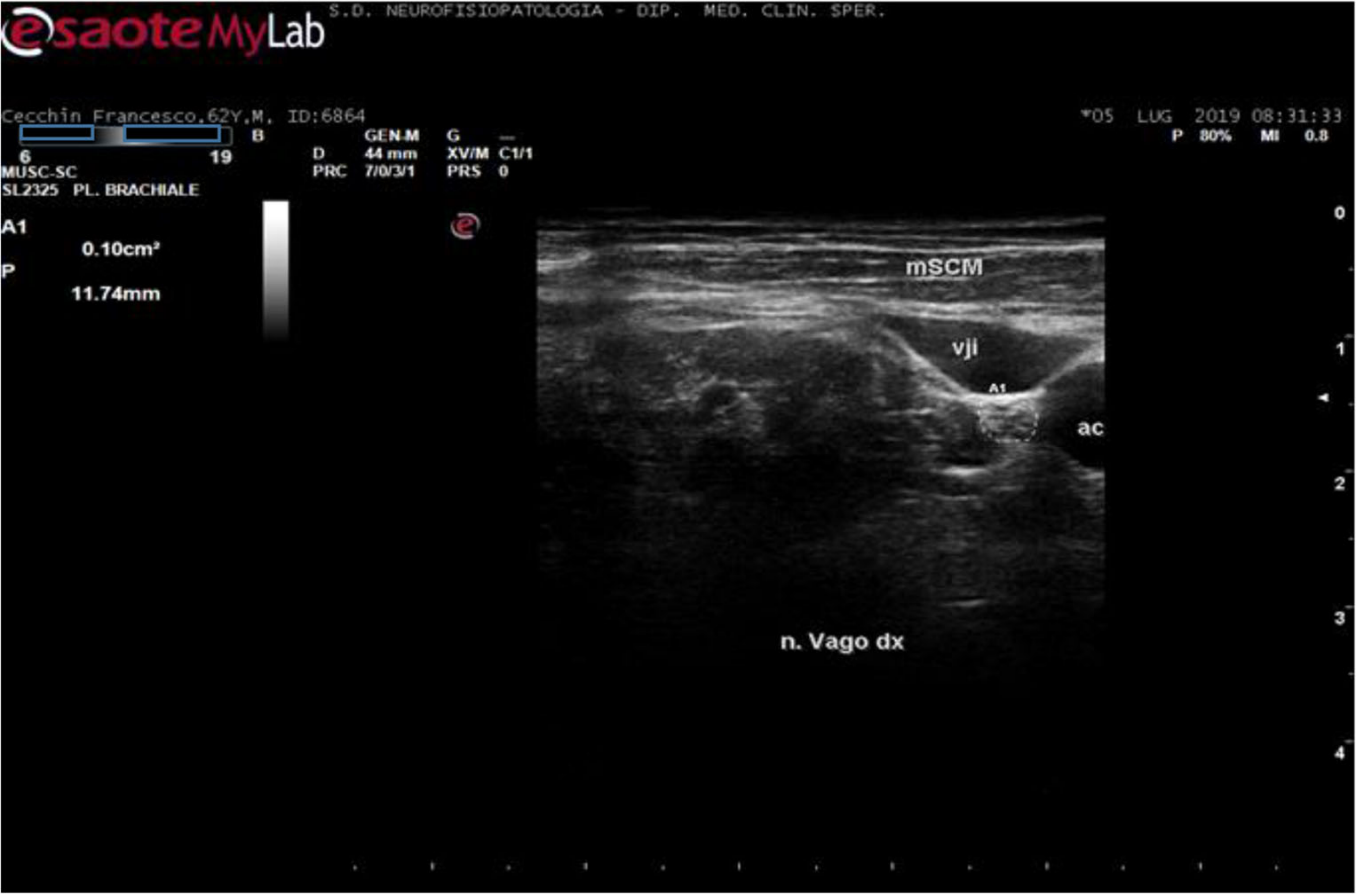
Typical axial high-resolution ultrasonography (HRUS) of short-axis view of the right VN (inside the dotted line) in a healthy control subject (C.F., M, 62 years). mSCM, sternocleidomastoid muscle; vji, internal jugular vein; ac, carotid artery; dx (destri), right side

**Fig. 2:**
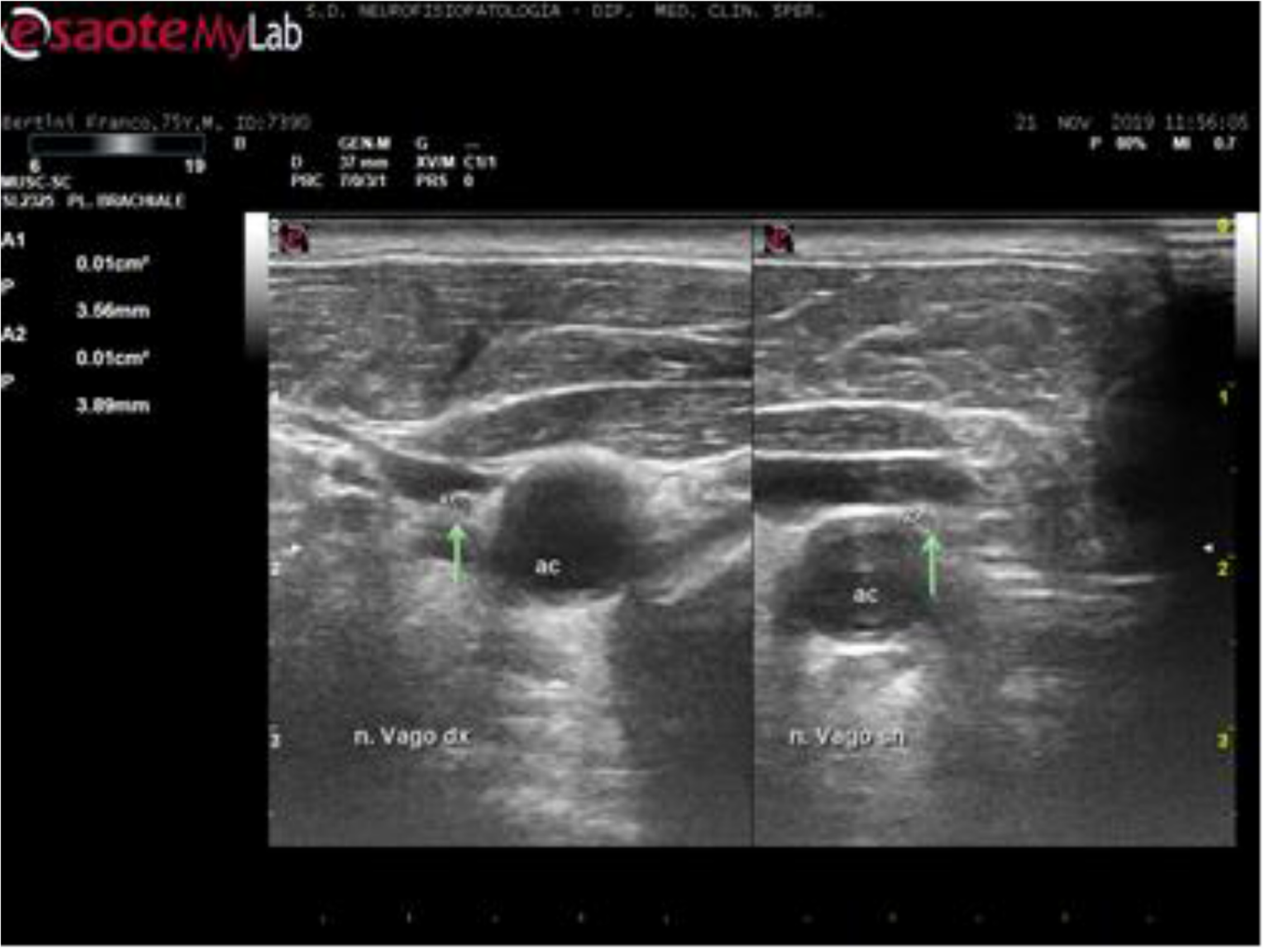
Typical high-resolution ultrasonographic findings of VN on both sides (arrow) in a patients with Parkinson’s disease (case BF, M, 75 years old, disease duration 9 years, hyperkinetic–hypertonic type). Note the substantially symmetric clearly reduced CSA of VN and the presence of a thin hyperechoic rim and no appreciable internal structure. ac, carotid artery

Participants were scanned lying in the supine position, with slight head extension while turning his/her head to the side opposite to the scanned nerve. The VN was scanned in the axial view; individually optimized settings were used for each subject with respect to gain, depth, and focus. Depth was set at 4 cm, and the probe was placed first at the level of the thyroid lobe with the probe orientation marker directed toward the patient’s right side. The probe was then moved laterally to identify the nerve inside the carotid sheath. Both the carotid artery and the internal jugular vein served as anatomical landmarks [32]. The VN was identified as a small rounded hypoechoic/or honeycomb structure widget deep to the carotid artery and jugular vein (see Fig. 1 for an example in a control subject). Minimal pressure was applied during the examination in order to prevent nerve compression. We measured the CSA of the VN by following the contour of the nerve just inside the hyperechoic rim with the probe exactly orthogonal to the nerve and with least pressure applied; in addition, we considered the perimeter, by tracing the nerve following the hyperechoic epineurium, and only qualitatively echogenicity and structure of the VN trunk. In the same session, the sural and tibialis nerves were also scanned following the commonly used modalities [19, 33] to exclude a possible diffuse peripheral neuropathy. (Fig. 2)

#### Statistical analysis

Statistical analysis was performed using SigmaPlot version 12.0 package (Systat Software Inc., 2011–2012). Data were tabulated, calculated, and analyzed; the mean and SD of VN CSA and perimeter were determined. The measure obtained from the left side in controls and patients was used in the correlation and comparison studies. Two-tailed Student’s test or Mann–Whitney rank sum test was used to compare sonographic measure between controls and patients, side-to-side measures, and subgroups of patient findings.

Pearson correlation coefficient (Pearson product-moment correlation) was employed to correlate VN ultrasound measurements with age, weight, height, BMI, and disease duration.

## Results

The main clinical features and HRUS findings we detected in patients, together with control demographic data, are summarized in Table 2. Patients and healthy control subjects were well balanced in terms of main features. No signs of peripheral neuropathy were present in either sample; trunk dimension and the structure investigated in the sural and tibialis posterior nerve on the lower left limbs resulted within normal limit (CSA in IPD, sural nerve =4.9 + 1.7 mm^2^; tibialis posterior nerve 12.4 + 4.3 mm^2^; in healthy controls, sural nerve 5.3 + 1.4; tibialis posterior 13.4 + 4.1; *p* value not significant). Furthermore, no difference in BMI or other anthropometric data was observed.

**Table 2:** Features of sonographic measurements in IPD patients compared with controls

	Controls (*n* = 20)	IPD patients (*n* = 20)	*p* value
VN CSA (mm^2^)	R 6.0 + 1.33 L 5.6 + 1.26	2. 37 + 0.91 1.87 + 1.35	< 0.001 < 0.001
VN perimeter (mm)	8.87 + 0.86 L 8.58 + 0.97	5.06 + 0.85 4.78 + 1.74	< 0.001 < 0.001
Body mass index	24.47 + 4.95	21.06 + 3.88	n.s.
Echo structure	Round hypoechoic honeycomb: 420	Round hypoechoic 40 (with rim 6)	
Sural nerve CSA (mm^2^)	5.3 + 1.4	4.9 +1.7	n.s.
Tibial nerve CSA (mm^2^)	13.4 + 4.1	12.4 + 4.3	n.s.

In both sides, the VN CSA was significantly smaller in IPD outpatients (right 2.37 + 0.91 mm^2^, left 1.87 + 1.35 mm^2^) than in controls (6.0 + 1.33 mm^2^, 5.6 + 1.26 mm^2^; *p* <0.001). Also the perimeter resulted less (right 5.06 + 0.85, left 4,78 + 1.74 mm) compared with controls (8.87 + 0.86 mm, 8.58 + 0.97 mm; *p* <0.001). No significant differences in the right or left were detected in our sample. Honeycomb structure was lost or not appreciable.

There were no significant correlations between VN CSA and age, the Hoehn and Yahr scale, disease duration and severity, and therapy.

## Discussion

Our findings provide evidence that IPD is associated with bilateral atrophy of the VNs in the absence of signs of peripheral neuropathy and that can be detected in vivo by HRUS. In fact we found that both VNs are significantly thinner in IPD compared with healthy normal controls, in agreement with other authors [15, 25, 26]. No potentials influenced of any comorbidity associated with VN neuropathy may be called in question because they have been preliminarily excluded before patient inclusion in the study. As regards the inner structure, the nerve was hypoechoic with or without a detectable rim; the fascicular pattern was lost or not evident.

A systemic peripheral neuropathy, previously reported in IPD, which might also involve the VN [34], can be excluded by normal electrophysiological findings and HRUS in both the sural and tibialis posterior nerves.

No correlation of VN calibers with IPD age, disease duration, and severity, nor with levodopa dose, was observed; moreover, VN atrophy seems unlikely related to neuropathy associated with levodopa medication [35].

Similar or even more pronounced VN atrophy has been reported also in patients with diabetic neuropathy [18, 19] and ALS [20]; furthermore, HRUS are able to discriminate involvement also in small nerves [19, 33].

The VN as has been hypothesized is involved in IPD progression, following an active retrograde transport of α-synuclein coming from the enteric nervous system and ascending the nerve until reaching the DMVN in the brainstem and cortex [4, 36]. Its role is further confirmed by the decreased risk of disease following sub-diaphragmatic truncal vagotomy [9, 37]. The transport via the vagal trunk to the DMVN has been confirmed in animal in which α-synuclein, recombinant or derived from human PD brain lysate, has been injected into the intestinal wall and then the substance has been found in section of VN of IPD patients [7, 38, 39]. Therefore, a some degree of VN atrophy is for nothing no surprising by keeping in mind the high sensitivity of this long nerve to α-synucleinopathies even if does not prove an ascending IPD pathology from the gut via VN [26].

Taking into account that the VN at cervical levels contain unmyelinated visceral sensory fibers ascending mainly to the nucleus of the tractus solitarius (NTS), unmyelinated or partially myelinated visceromotor and cardio-inhibitory fibers stemming in the DMVN, and thick myelinated somatomotor fibers coming from the nucleus ambiguous; that in IPD, neural degeneration with Lewy bodies has been found in the DMVN and NTS but not in the nucleus ambiguus and the somatosensory nucleis, and that the number of unmyelinated fibers is about four times higher than that of myelinated fibers in the VN [40–42], we can hypothesize mainly a loss of unmyelinated fibers [26]. Further studies using very high US (70 Mz) might help to further improve the accuracy of fascicular involvement [43].

There are a handful of studies investigating VN size and morphology in IPD in vivo. Our results confirm VN nerve atrophy reported by other authors [15, 25, 26]. Nevertheless, nonsignificant asymmetry was detected, in contrast with Peltz et al. [15]. However, they are contrary to those reported by two other studies [27, 28]. In the first paper, the AA measured the cross-sectional area and echogenicity without detecting any significant ultrasonographic changes of the VN integrity. Looking at their findings, we can note that the CSA mean values in their control group are a little smaller than that previously reported in other studies [18, 19, 44] and might partially account of their results. For the other paper, only the diameter was measured without considering the CSA commonly considered the most reliable measure to evaluate nerve integrity [25, 44]. Another consideration could be that our patients were in a more advanced stage of disease compared with the two cited works. Moreover, despite similar sample size of these studies with the present one, the parameters of the VN remain to be untangled, and studies with larger sample sizes and longer follow-up periods are necessary.

Our study has several limitations: firstly, the small sample of patients; secondly, the lack of assessment of autonomic function and so no correlation with the burden of autonomic symptoms can be drawn; and lastly, not less important, the noninclusion of patients at early disease’s stages or de novo patients. Thus, studies in larger samples of patients with IPD, including de novo cases, and other forms of parkinsonism are necessary to further elucidate the diagnostic value of HRUS of VN in this disease.

In conclusion, at this stage of investigation, there is a convincing body of evidence that HRUS of the VN represents a promising non-invasive in vivo imaging modality of screening VN in IPD patients independent of disease stage and duration and an interesting index to identify patients at risk or to confirm the disease and to develop new disease-modifying strategies.
